# Exploring perceptions and preferences for PrEP choice and of an mHealth intervention: insights from the ImPrEP CAB‐Brasil study

**DOI:** 10.1002/jia2.26493

**Published:** 2025-07-02

**Authors:** Cristina Pimenta, Claudio G. Mann, Brenda Hoagland, Eduardo Carvalheira, Cristina Jalil, Marcos Benedetti, Nilo Fernandes, Carolina Coutinho, Emilia M. Jalil, Mayara Secco Torres Silva, Roberta Trefiglio, Alessandro Farias, Maria Paula G. Mourão, José Valdez Madruga, Josué N. de Lima, Ronaldo Zonta, Gabrielle O'Malley, Valdilea G. Veloso, Beatriz Grinsztejn, Thiago S. Torres, for the ImPrEP CAB‐Brasil Study

**Affiliations:** ^1^ Instituto Nacional de Infectologia Evandro Chagas, Fundação Oswaldo Cruz (INI‐Fiocruz) Rio de Janeiro Brazil; ^2^ Centro Especializado em Diagnóstico, Assistência e Pesquisa ‐CEDAP Salvador Brazil; ^3^ Fundação de Medicina Tropical Heitor Vieira Dourado Manaus Brazil; ^4^ Centro de Referência e Treinamento em DST/Aids ‐ CRT São Paulo Brazil; ^5^ Centro de Referência em DST/Aids Campinas Brazil; ^6^ Aconselhamento ‐ CTA/Policlínica Centro Florianópolis Brazil; ^7^ Department of Global Health Schools of Medicine and Public Health, University of Washington Seattle Washington USA

**Keywords:** HIV prevention, PrEP, implementation, mHealth, cabotegravir, sexual and gender minorities

## Abstract

**Introduction:**

Although the efficacy of long‐acting injectable cabotegravir (CAB‐LA) for pre‐exposure prophylaxis (PrEP) is well‐known from clinical trials, research is needed to guide effective strategies for its implementation. We describe a qualitative study to assess perceptions and preferences for PrEP choice and acceptability of an mHealth intervention within the ImPrEP CAB Brasil study.

**Methods:**

ImPrEP CAB Brasil is an implementation study of same‐day delivery of CAB‐LA for young sexual and gender minorities (SGM; 18–30 years) in oral PrEP public health clinics in six Brazilian cities. At enrolment, participants received counselling on HIV prevention (SOC) or SOC+mHealth tool to choose between oral or injectable PrEP. The mHealth tool consisted of five videos describing HIV combined prevention including PrEP options. A subset of participants from each site were invited to participate in the qualitative study (October 2023−July 2024). Semi‐structured interviews were conducted, recorded and transcribed. Data were fed into ATLAS.ti.24 software. Conventional content analysis was used for coding categories based on an inductive reasoning process.

**Results:**

We conducted 120 interviews (48 SOC and 72 SOC+mHealth; 107 CAB‐LA and 13 oral PrEP). Participants reported not knowing about CAB‐LA before enrolment; some recently heard from a partner or friend. Reasons for choosing CAB‐LA were perceived convenience, practicality, easier adherence to bimonthly injections and higher efficacy compared to oral PrEP. Reasons for not choosing CAB‐LA were fear of injections and pain. Reasons for choosing oral PrEP included perspective of less appointments, easiness of daily adherence, access in case of travel and the option to stop immediately if desired or needed. Reasons for not choosing oral PrEP included forgetfulness of daily intake, gastrointestinal side effects, fear of inadvertent exposure and judgement by family. Participants found the mHealth educational tool useful and adequate for PrEP education and decision‐making.

**Conclusions:**

Perceptions for PrEP choice among SGM underscore the importance of providing comprehensive information and support towards decision‐making processes, so users can have an accurate understanding of each PrEP option, as well as their clinical and social benefits. The mHealth tool was perceived as highly desirable and useful for PrEP education, having the potential to be implemented in HIV prevention services.

**Clinical Trial Number:**

NCT05515770

## 1 INTRODUCTION

The HIV epidemic in Latin America remains concentrated in vulnerable populations, with gay, bisexual and other men who have sex with men (MSM) and transgender women bearing the highest burden. Overall, regional data indicate a 9% increase in new HIV acquisitions from 2010 to 2023 [1]. While the estimated HIV prevalence in Brazil in 2023 for the general population is approximately 0.4%, it is significantly higher among MSM (23%) and transgender women (30%) [2, 3, 4]. Additionally, sexual and gender minorities (SGM) aged 30 years or younger are particularly at increased vulnerability to HIV. In the ImPrEP seroincidence study, HIV incidence was three times higher among Brazilian SGM not using pre‐exposure prophylaxis (PrEP) aged 18–24 years compared to those aged > 30 years [5]. Curbing the HIV epidemic requires scale‐up and consolidation of comprehensive HIV prevention programmes and increasing access to different prevention methods including PrEP.

The efficacy of long‐acting cabotegravir (CAB‐LA), a novel integrase strand‐transfer inhibitor administered as a gluteal intramuscular injection, has been well established through clinical trials, specifically HPTN083 and HPTN084 [6, 7]. As a result, the World Health Organization (WHO) recommended CAB‐LA for PrEP in July 2022 as part of the standard prevention regimen for individuals at increased vulnerability to HIV [8]. This recommendation reflects a growing global consensus on the necessity of diverse PrEP modalities, as CAB‐LA PrEP enhances protection with the potential to overcome adherence issues associated with daily oral PrEP [9]. By offering a range of preventive technologies, we can better meet the varied and evolving needs of individuals throughout their life span. This approach can significantly improve PrEP coverage by providing consistent protection through infrequent and discrete administration without disclosure, particularly among young SGM. The largest oral PrEP implementation study conducted in Latin America (ImPrEP) showed that MSM and transgender women of younger age had lower oral PrEP adherence compared to their older counterparts [10, 11]. The long‐acting PrEP formulation is seen as a particularly important option for young SGM and those most vulnerable populations who historically face barriers to consistent adherence to oral PrEP [12, 13].

Mobile health technologies (mHealth) have been created as strategies to support PrEP adherence and retention in PrEP services. Interventions with videos containing information on HIV prevention have shown effectiveness to increase PrEP uptake and adherence among MSM [14]. Numerous mHealth initiatives incorporate user‐centred modifications to be more attractive to the diversity of SGM populations [14]. However, these tools should be accessible and available to these populations to achieve effectiveness.

Implementation studies are essential to evaluate the effectiveness and feasibility of CAB‐LA for PrEP in real‐world settings, and to identify optimal delivery methods within a scalable framework. By understanding how to effectively integrate CAB‐LA into existing healthcare systems, we can enhance HIV prevention efforts and ultimately reduce HIV incidence. In Brazil, while CAB‐LA has been registered and approved for PrEP by the national regulatory agency (ANVISA), it is yet not accessible through the Brazilian Public Health System.

The ImPrEP CAB Brasil study aims to generate critical evidence to inform national policies and programme implementers on optimizing the delivery of CAB‐LA within public health PrEP services in a context of choice (CAB‐LA or oral PrEP) for young SGM aged 18–30 years. In this manuscript, we explore perceptions and preferences for PrEP choice and for an mHealth intervention tool among a subset of participants of the ImPrEP CAB Brasil study.

## 2 METHODS

### 2.1 ImPrEP CAB Brasil study

ImPrEP CAB Brasil is a type 2 hybrid effectiveness‐implementation study designed as an open‐label cohort with a convergent mixed methods approach, incorporating both quantitative and qualitative data [15]. Eligibility criteria included: (1) age 18–30 years; (2) self‐identification as cisgender MSM, nonbinary person assigned male at birth, transgender woman or transgender men; (3) attend the service seeking PrEP; (4) PrEP‐naïve; and (5) negative HIV test results. Details of the study protocol are published elsewhere [16]. Briefly, individuals seeking PrEP in one of the six PrEP services in six Brazilian cities (Rio de Janeiro, São Paulo, Manaus, Salvador, Florianópolis and Campinas) were invited to participate in the Step 1 study phase when they could choose between oral or injectable PrEP. Additionally, participants received (1) standard of care (SOC) counselling covering HIV combination prevention including oral PrEP and CAB‐LA or (2) SOC+mHealth education intervention. Of 1447 participants included in Step 1, 1200 (83%) chose CAB‐LA, and those exposed to SOC+mHealth were over twice as likely to choose CAB‐LA compared to SOC [17]. Participants opting for CAB‐LA were enrolled in the Step 2 phase and were followed for at least 52 weeks (bimonthly visits).

**Figure 1:**
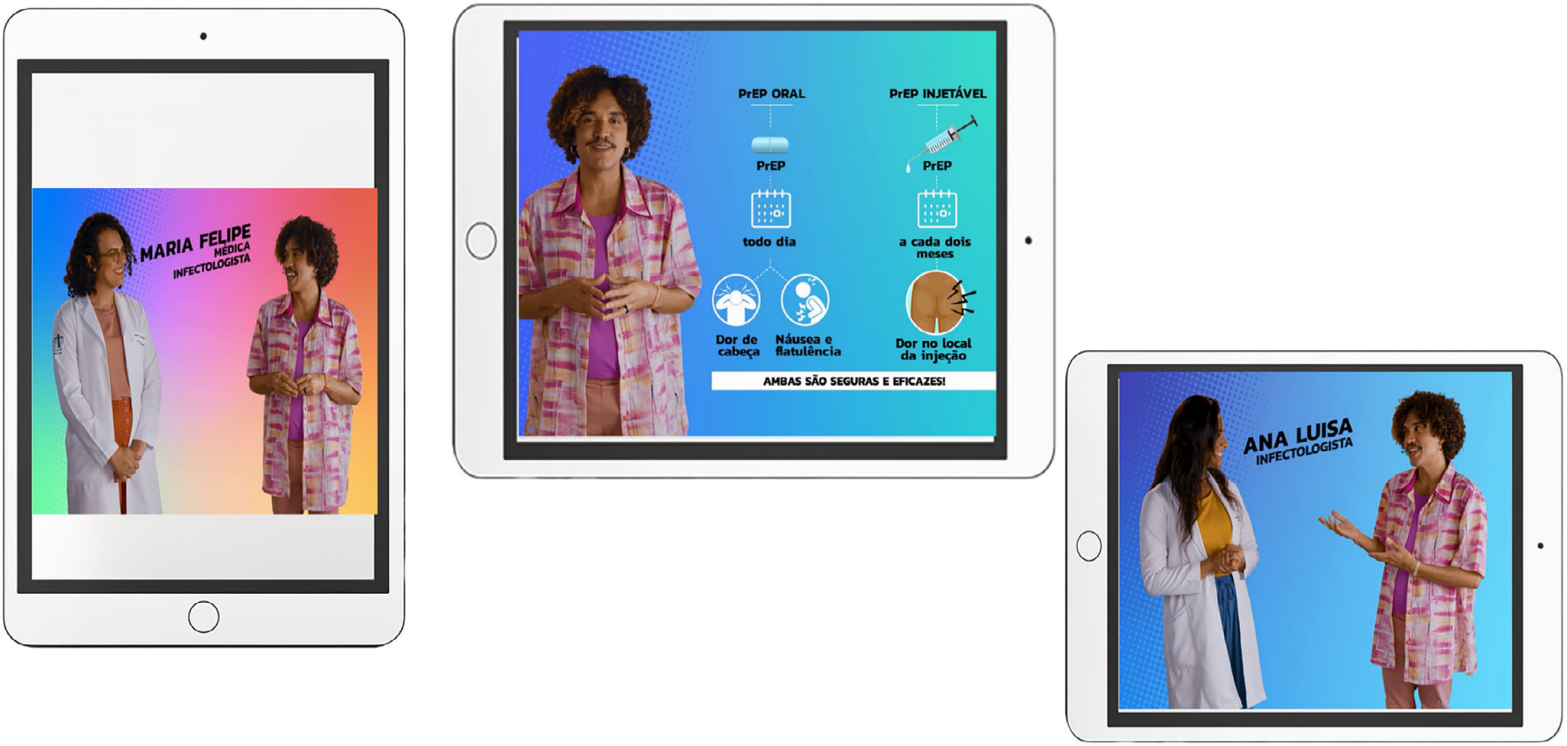
**The mHealth tool. Prints from the videos**. Abbreviations: PrEP, pre‐exposure prophylaxis.

The mHealth intervention comprised five videos (Figure 1) presented on a tablet (17 minutes duration). The videos, with subtitles in Portuguese and sign language, were designed to provide information on: oral and injectable CAB‐LA PrEP as prevention tools; oral PrEP, adherence and side effects; CAB‐LA PrEP, adherence and side effects; HIV post‐exposure prophylaxis and testing for sexually transmitted infections, under a combined prevention approach (Supporting Information 1). Participants could watch the videos in privacy. Detailed information on the development of the mHealth tool is described elsewhere [18].

### 2.2 Ethical considerations

This study was reviewed and approved by the Evandro Chagas National Institute of Infectious Diseases–Fiocruz institutional review board (#CAAE 59166522.7.1001.5262), the WHO Research Ethics Review Committee and local institutional review boards at each site. All participants provided written informed consent (no illiterate participants were included). All methods were carried out in accordance with relevant guidelines and regulations. This study was registered at ClinicalTrials.gov (NCT05515770) on 29 August 2022.

### 2.3 Qualitative study procedures

We conducted a qualitative study aligned to the ImPrEP CAB Brasil objectives and eligibility criteria to gain in‐depth insights on perceived advantages and disadvantages about PrEP choice preferences. Participants who received SOC+mHealth were also asked about their perceptions of the mHealth intervention. We followed consolidated criteria for reporting qualitative research (COREQ) (Supporting Information 2) [19].

A subset of participants included in Step 1 were purposively invited to participate in individual semi‐structured interviews conducted by a team of 12 experienced qualitative interviewers at each study site (male and female psychologists and counsellors). Interviewers did not participate in coding and analysis. An interview guide to elicit insights and perceptions was developed for participants who received SOC and SOC+mHealth (Supporting Information 3). Participants were approached face‐to‐face and invited with no refusals. Participation was voluntary and their PrEP choice was assured regardless. Interviews were conducted in private to facilitate open sharing, ranging from 15 to 30 minutes. They were audio‐recorded and transcribed verbatim throughout the study process. Researchers had no relationship with participants prior to the interview. The role of the researchers was multifaceted, encompassing responsibilities as coders and analysts. Personal identifiable information inadvertently mentioned during the interview was removed during the transcription process. We used study participant numbers to identify transcribed interview files. Two qualitative team researchers reviewed the first three interview recordings from each site for quality control. There were no repeat interviews. Saturation was considered without modifications to the interview guide.

We used conventional content analysis as articulated by Bardin and Hsieh, which uses an inductive, flexible approach to qualitative data analysis, focused on the emergence of themes from the data, the systematic organization of content through coding, and the importance of context in interpreting findings [20, 21]. Qualitative analysis of PrEP choice was assessed through thematic descriptions of interviews that convey participants’ perceptions and understanding of the advantages and disadvantages of PrEP options, and the reasons for their choice. Content analysis from participants receiving the mHealth intervention was used to generate a thematic description of acceptability [22, 23].

The research team performing the qualitative analysis included experienced PhD social scientists (male and female psychologists, sociologists, public health specialists) highly experienced in qualitative methodologies. An initial codebook was developed by two researchers (CGM and EC) and evaluated for coherence by different researchers (CP and NF). Transcribed texts were reviewed and validated by two interviewers (DMW and JRG) prior to coding and analysis. Code labelling and units of analysis were identified by iteratively examining the texts. Two qualitative researchers (CGM and EC) independently coded six transcripts using the initial codebook and added additional inductive codes. Researchers (EC, CJ and NF) exchanged and reviewed the coded transcripts, noted and discussed discrepancies in codding interpretation until a consensus was reached. Qualitative analysts (CP, CGM, EC and NF) split and coded the remaining transcripts, proposing additional codes as necessary. Codes and transcripts were migrated to ATLAS.ti Scientific Software Development GmbH (version 24) in Portuguese, to aid data management and processing through simultaneous collaborative work. The final codebook was consolidated and used to code all transcripts. Preliminary thematic analysis was conducted by three researchers and reviewed by the larger study team and site‐level collaborators for consistency and validity of interpretation. When synthesizing findings, we intentionally endeavoured to balance interpretations between individual psychological processes and social contextual factors by engaging in peer debriefing and feedback from colleagues ensuring that multiple perspectives were considered.

## 3 RESULTS

From October 2023 to July 2024, a total of 120 interviews were conducted among 48 (eight per site) participants who received SOC and 72 (12 per site) who received SOC+mHealth. Most participants were cisgender men (85.0%), aged 25–30 years (58.4%), Black or *Pardo* (59.1%), had initiated or completed tertiary education (65.8%), chose CAB‐LA PrEP (89.2%) and received SOC+mHealth (60.0%) (Table 1). Among those choosing CAB‐LA, 42 (39.3%) were exposed to SOC and 65 (60.7%) to SOC+mHealth (Table 1).

**Table 1:** Characteristics of interviewed participants

	Total (*N* = 120; %)
**Age (years)**	
18−20	12 (10.0)
21−24	38 (31.6)
25−30	70 (58.4)
**Gender**	
Cisgender man	102 (85.0)
Transgender woman	11 (9.2)
Transgender man	4 (3.3)
Nonbinary assigned male at birth	3 (2.5)
**Race**	
Asian	1 (0.9)
Black	33 (27.5)
Indigenous	1 (0.9)
*Pardo* (mix‐race)	38 (31.6)
White	47 (39.1)
**Education**	
Primary incomplete	1(0.9)
Primary complete	1 (0.9)
Secondary incomplete	5 (4.1)
Secondary complete	34 (28.3)
Tertiary incomplete	41 (34.2)
Tertiary complete	38 (31.6)
**PrEP choice**	
Oral	13 (10.8)
Injectable	107 (89.2)
**mHealth tool intervention**	
Yes	72 (60.0)
No	48 (40.0)

Abbreviations: mHealth, mobile health; PrEP, pre‐exposure prophylaxis.

**Figure 2:**
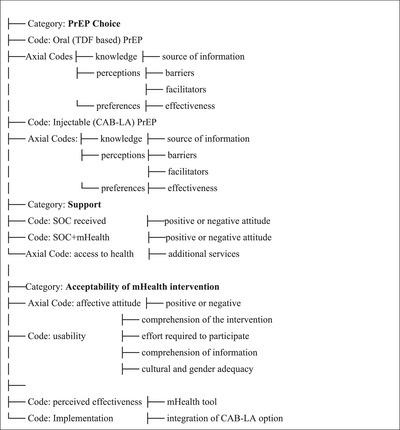
**Coding Tree—factors influencing PrEP choice**. Abbreviations: CAB‐LA, cabotegravir long‐acting; PrEP, pre‐exposure prophylaxis; SOC, standard of care; TDF, tenofovir disoproxil fumarate.

Data were organized under three major categories (PrEP choice, support, acceptability of the mHealth intervention). Additional axial codes were inductively derived from the transcribed text reports (oral and injectable PrEP with CAB‐LA, knowledge, perceptions, preferences, counselling; affective attitude usability associated with acceptability; and implementation associated with both CAB‐LA PrEP and the mHealth tool). Code labelling and units of analysis were identified by iteratively examining the texts and isolating self‐contained segments of information and insights from transcripts of each study arm (SOC and SOC+mHealth) allowing a more integrated understanding of the data (Figure 2).

Thematic analysis and contextual understandings are presented considering participants’ knowledge, perceptions, preferences and understandings of the factors influencing PrEP choice, support, acceptability of the mHealth intervention and implementation as follows.

### 3.1 PrEP choice

Content analysis of factors influencing PrEP choice explored if the participant had acquired knowledge before coming to the service, including awareness of both PrEP modalities (oral and CAB‐LA) at the day of inclusion, their previous sources of information and perceived preferences. Most participants reported having heard about oral PrEP and not knowing about long‐acting injectable PrEP before enrolment.

I asked some people who had been using it for a while and learned that it is a drug in pills that will really protect you against HIV; not against other STIs though. (Cis MSM, age 18, SOC+mHealth arm, injectable PrEP)

I had searched the internet, I knew about the existence of oral PrEP, I knew about the pill. But before coming here I didn't know there was an injectable form. So, before the study I thought it was just the pill. (Cis MSM, age 27, SOC+mHealth arm, injectable PrEP)

Motivation for seeking PrEP and choosing either PrEP modality was related to participants’ risk perception of acquiring HIV. PrEP choice was influenced by participants’ preferences based on interpretations of facilitating factors and barriers, perceived effectiveness of the product and understanding of known side effects, as well as perceived practicality.

In fact, I'm here because I realized that in most of the relationships I have, people never want to use condoms. (Trans woman, age 24, SOC arm, injectable PrEP)

I have a hard time taking daily medications because I may forget and be unprotected. For that reason, I have chosen the injectable to make it easier. (Cis MSM, age 22, SOC arm, injectable PrEP).

I have a partner who has HIV but is undetectable. We wanted to be safe. He said the public health service had PrEP and that you are well assisted. I came here seeking to be informed precisely to be able to start. (Cis MSM, age 28, SOC+mHealth arm, injectable)

Facilitating factors associated with choosing oral PrEP included being familiarized with the PrEP modality, knowing more about this option and having acquaintances that already use it. Additional reasons described were the perspective of having less return visits for clinical appointments (every 3 months), easiness of daily pill adherence, not using injections, easy access in case of travel and perceiving as an option that you can stop immediately if desired or needed.

I chose oral PrEP because you don't have to use an injection. You can take it at home, you don't have to go anywhere, and less frequent clinical visits. And for me I believe that it literally fits. (Cis MSM, age 27, SOC+mHealth arm, oral PrEP)

Even though some participants said they found injectable PrEP more interesting and advanced, they reported opting for oral PrEP at this time due to its wider availability and well‐known side effects, and that they might consider switching to the new PrEP modality in the future.

I opted for oral PrEP because I believe I'll be able to correctly accomplish taking the pills every day. Injectable PrEP would be interesting too. But I have a lot of, difficulty with needles. And I don't know that much about it. It is a new medication. I prefer oral PrEP, which has been used longer. If it's available in the future I may change. (Cis MSM, age 28, SOC+mHealth arm, oral PrEP)

Conveyed barriers for not choosing oral PrEP and initiating injectable PrEP included the risk of not adhering properly and reporting forgetfulness of daily intake. Not having an adequate private place to store the medication along with fear of inadvertent disclosure of daily pill and consequent judgement by family members and partners was also a concern. The possibility of switching PrEP modalities if desired or needed seems to make the choice between options more comfortable.

I think forgetting to take a pill is something that can happen easily. So, I feared to start PrEP and end up not having adequate adherence. So, when this injectable option was offered to me it seemed very interesting. The best option. (Cis MSM, age 27, SOC arm, injectable PrEP)

…For my family my sexuality is still a taboo. They could question the use of the drug if they saw it. …I think the injectable now makes me more comfortable, but in the future, I can change. (Cis MSM, age 27, SOC arm, injectable PrEP)

Reasons for choosing CAB‐LA included perceived convenience and practicality, easy adherence to bimonthly injections, especially for those who have busy routines and propensity for forgetfulness. Perceived higher efficacy and less concerns about interruptions compared to the daily oral option was also pointed out. Most understood that CAB‐LA has fewer known side effects and conveyed feelings of greater confidence, security and protection without the need to use a daily oral medication. For those who chose CAB‐LA, an injection every 2 months is seen as more convenient and effective with less social stigma despite the discomfort with needles.

My biggest fear is that my routine will end up being busy because of college, work, and I end up forgetting. I could be exposing myself to the virus. So, for me, I think the injectable would be the best option. (Cis MSM, age18, SOC+mHealth arm, injectable PrEP)

I think the injectable is more practical. It is once a month at the beginning and every two months after, which I think makes the person much safer and easier to have protection. (Cis MSM, 18 years old, SOC arm, injectable PrEP)

I prefer injectable because it's practical and faster. When I take hormone pills, I hate it. Injectables, I take them and that's it; it's there in my body. This practicality is easier for me. PrEP pills give reactions such as nausea and the injection pain. (Trans woman, age 24, SOC+mHealth arm, injectable PrEP)

The underscored barrier to CAB‐LA was fear of needles and injections, apprehension of after‐pain and possible reactions. Other concerns were related to the longer duration of clinical visits and possible disruption of daily routines, especially work commitments. Suspicions of unknown long‐term effects of the long‐acting medication and fear of developing drug resistance were also expressed. Overall, perceived advantages seemed to outweigh the difficulties, and most participants perceived no barriers to using injectable PrEP.

I believe that my fear of needles is really the biggest reason. I don't like needles. But it is comforting to know that I won't be taking oral medication for ever and could eventually switch. (Cis MSM, age 29, SOC arm, injectable PrEP)

I think the challenge is to see how it will be after the injection. How much pain, swelling. I think it's more challenging to be able to continue using this method. That's my concern. But other than the pain, I think it's fine. (Cis MSM, age 28, SOC arm, injectable PrEP)

The only concern I have is about resistance, you know? The possibility of creating resistance in relation to the long tail of the medication. But I know it's something rare, difficult to happen. (Cis MSM, age 27, SOC arm, injectable PrEP)

No significant differences by age range and gender were found under PrEP choice. Considering initial perceptions of barriers to CAB‐LA use, we found that the older age group (25−30 years) tended to know more about oral PrEP and seemed to be more confident of their initial choice than the younger group (18−20 years). Transgender women experienced in injectable hormone use tended to consider CAB‐LA as an easier choice.

### 3.2 Support

Under support, we explored participants’ satisfaction with the quantity and quality of information and counselling received preceding opting for a PrEP modality, regardless of the mHealth intervention. We explored how well participants felt informed about their options, clarity of the information provided, and the overall effectiveness of the counselling sessions and the mHealth educational intervention.

Participants who underwent SOC expressed satisfaction and full understanding of the counsellors’ explanations about oral and injectable PrEP. They had no significant doubts and felt ready to adopt one of the options provided. They highlighted the clarity and dynamism of several health professionals when addressing the subject, helping them become more confident of their choice, and praised the care and welcoming of the health team. All participants said they had their doubts answered.

From the moment I went into the reception I felt care and zeal of the professionals, with nothing to complain about. Obviously, places that have a lot of demand, have some waiting…They provided all the information I needed and answered my questions, and I could see that they care for the patients. (Cis MSM, age 29, SOC arm, injectable PrEP)

The counsellor was careful to make everything very clear. The doubts I had were elucidated, and I felt they were solved. He managed to explain everything to me properly. (Cis MSM, age 22, SOC arm, injectable PrEP)

Most participants exposed to SOC+mHealth intervention valued the combination of face‐to‐face guidance with the video resource, where they felt the counsellor could focus on clarifying their doubts about the benefits and risks of oral and injectable PrEP, its administration and side effects, other sexually transmitted infections (STI) testing, clinical procedures and consultations, expected periodicity of return visits, and the possibility of switching in the future.

Since I had never heard of injectable PrEP, nor used oral PrEP, I had a series of questions. The counsellors’ explanation was very coherent, very clear. She clarified my doubts about the periodicity of injections. Also, about the lab exams, if they are monthly or at the following PrEP application. (Cis MSM, age 29, SOC+mHealth arm, injectable PrEP)

I understood everything. I think the video saves time. The videos explained most of the information about PrEP options and the professional clarified my doubts. So, I was able to get all the information in a comprehensive way. (Cis MSM, age 21, SOC+mHealth arm, injectable PrEP)

Some participants felt no need to have a counselling session focused on PrEP education and HIV prevention after the mHealth intervention, expressing feelings of the repetitiveness of information and boredom. However, they also pointed out the importance of having a health professional for emotional support and referral to additional healthcare services.

Everything was well explained, even repetitive, so that there were really no doubts. I asked the counsellor about other health concerns that I had, and she told me I can be assisted here as well. (Cis MSM, age 27, SOC+mHealth arm, oral PrEP)

It's a great method to apply… but I think that, from the moment I have a doctor here giving me the information, I don't need to see a video. … The video is good, it's didactic, it's practical, it's fast, it's illustrative but you don't need both. (Trans woman, age 24 SOC+mHealth arm, injectable PrEP)

### 3.3 Acceptability of mHealth

We assessed acceptability associated with participants’ perceived relevance and satisfaction with the mHealth intervention. Participants comprehended and praised the mHealth intervention highlighting its clarity, objectivity, practicality and interactive features. The usability of the tablet has been seen as appropriate, easily manageable requiring minimum effort and facilitating access to information. The segmentation of themes was viewed as advantageous for understanding. The use of simple, direct language and didactic approach was praised. The accessibility of information for different audiences including persons with hearing impairment was positively mentioned. Most participants expressed approval and satisfaction with the mHealth intervention.

I think it saves time for the professionals to work. I think it's a usable tool. This tool is more dynamic than just watching a professional talking. You have the information on the screen, the presenter's dialogues with the doctors, and the captions if you need. The interaction really draws the persons’ attention to the subject. (Cis MSM, age 26, SOC+mHealth arm, injectable PrEP)

I think it works well because it brought me greater security. It helped to reinforce the information. The videos were well divided, very didactic. Even regarding the negative effects, and positive ones. It reinforced my desire to use the injectable method. (Cis MSM, age 21, SOC+mHealth arm, injectable PrEP)

Participants’ insights indicated they found the mHealth interesting and dynamic. In general, the mHealth tool was viewed as very useful, well‐designed and suitable for different audiences, particularly young people. They praised the effective communication and easy understanding provided by the digital tool and considered it valuable for education and informed decision‐making about PrEP.

Insights were given for wider dissemination of mHealth in support of injectable PrEP including the use of social media, television networks, schools and social encounter places for the LGBTQIAPN+ community in support of CAB‐LA implementation at existing oral PrEP public health services.

This material could even be shown to adolescents. Ways to communicate HIV and STI prevention, like the mHealth videos would be wonderful as informative material on the internet and other social networks, LGBTQ+ community centres and schools. (Cis MSM, age 27, SOC+mHealth arm, injectable PrEP)

The mHealth videos for prevention could be disseminated. I think it's essential to expand the use of the videos in other digital platforms and at gay clubs and bars. (Cis MSM, age 26, SOC+mHealth arm, injectable PrEP)

I think it's a matter of greater visibility for implementation. We could have access to the information before coming here to the health care service, for example. Not just within the services. (Cis MSM, age 24, SOC+mHealth arm, injectable PrEP)

## 4 DISCUSSION

Here, we explored perceptions and preferences for PrEP choice and of an mHealth intervention among participants from the ImPrEP CAB‐Brasil study. Perceived risk of not adhering properly to daily oral medication was seen as a major barrier to choosing oral PrEP, especially for those who have busy routines and a propensity to forget, whereas fewer returns for appointments, easiness to take medicines anywhere, not taking injections and the option to stop use if needed or desired were perceived as major advantages. While convenience, practicality, easy adherence to injections and perceived higher efficacy compared to daily oral PrEP were major reasons for choosing injectable PrEP, fear of injection and pain were the most relevant barriers for not choosing injectable PrEP. We found that differences in PrEP choices were primarily linked to contextual life factors associated with practicality, discretion, disclosure, stigma and possible side effects as opposed to gender and age preferences among young SGM persons interviewed.

In agreement with previous studies from Brazil on PrEP preferences, the perceptions of young SGM regarding the effectiveness and side effects of PrEP options ascertained the importance of providing detailed information of each PrEP product available for adequate literacy in support of PrEP choice [24, 25]. This detailed information is essential for fostering adequate knowledge and supporting informed PrEP choices. Furthermore, this research has demonstrated a strong desirability for the injectable PrEP option. These findings underscore the importance of PrEP literacy, which includes an accurate understanding of HIV risk for proper method adherence, as well as its clinical, biological and social benefits. An earlier qualitative study with key SGM informants experienced in oral PrEP from five Brazilian cities indicated the need to expand PrEP communication for greater accessibility, especially among populations in situations of greater vulnerability, with appropriate language for these groups [26]. The complementarity between prevention technologies was also highlighted as a great advantage, as it allows users to opt or combine methods according to their present lifestyle [26, 27].

We found that SOC counselling combined with the mHealth tool was significantly effective in delivering comprehensive information about PrEP modalities, including their safety and efficacy. This approach not only enhanced understanding but also empowered individuals to make informed choices among the available options. In a study that explored the acceptability of PrEP among SGM persons of migrant communities in Scotland, they found the need to identify appropriate communication methods in the context of diverse HIV literacy and address HIV risk concerns for PrEP uptake [27].

Previous studies have shown the efficacy and acceptability of mHealth tools and other digital health interventions for HIV prevention among young SGM [13, 28–30]. Our findings revealed that young SGM persons in Brazil not only praised the use of the mHealth intervention for its comprehensive information about PrEP and other HIV prevention methods but also valued the visual engagement through tailored cultural and gender‐appropriate characters, which promoted increased attention and satisfaction with the mHealth tool. However, participants’ perception of the effectiveness of the mHealth intervention in supporting PrEP education and choice in a context of integrated public health PrEP services, notably showed that the mHealth tool may not replace SOC with the professional as a stand‐alone intervention. Nevertheless, it could be implemented as a self‐education tool that makes the process more dynamic, leads to greater interest from persons seeking PrEP and positively influences PrEP uptake.

Further qualitative studies are essential to gain a deeper understanding of the contextual factors and lifestyle circumstances that may contribute to injection interruptions, delays in returning for injection appointments, as well as overall persistence and adherence. It is also important to explore perceptions and motives behind switching between PrEP modalities. Addressing these knowledge gaps will contribute to better inform strategies to enhance the adequacy and effectiveness of providing PrEP options and ultimately improving outcomes for persons most vulnerable to HIV.

This study has limitations. Although qualitative studies are essential to provide information and meaningful insights of participants’ perceptions, feelings and attitudes, allowing the understanding of the contextual factors that influence data, their subjective nature can lead to potential biases in coding and interpretation, as researchers may inadvertently impose their own perspectives on the data. To mitigate potential bias, we ensured a rigorous iterative analysis process among experienced qualitative researchers with careful consideration to the balance of depth of analysis with practical constraints to enhance the consistency and precision of the study. Another limitation is that a greater proportion of overall study participants chose CAB‐LA PrEP and most interviewees were from the CAB‐LA arm. Furthermore, although we included young SGM groups from six large Brazilian cities, the generalizability of findings may not be applicable to broader populations or different contexts.

## 5 CONCLUSIONS

Reasons for PrEP choice among young SGM point to the importance of providing comprehensive information and support in decision‐making processes, so users can have an accurate understanding of each PrEP modality and their clinical and social benefits. Daily oral and long‐acting injectable CAB‐LA are perceived as good options for PrEP among young SGM from Brazil. PrEP choice has been motivated by perceived vulnerability to HIV and perceived level of satisfaction or discomfort with the available options. Information on CAB‐LA is still narrowly disseminated, but stands out for its practicality and effectiveness, facilitating adherence to prevention and privacy of use. Interest and pursuit of oral PrEP was driven by knowledge gained from social media and reports from friends and sexual partners. The mHealth tool was perceived as highly desirable and useful for PrEP education. Our data may be used to inform health policymakers to develop strategies to support PrEP uptake and adherence. Programme implementers should consider mHealth interventions as an acceptable and feasible mechanism to strengthen and expand HIV prevention and PrEP education.

## COMPETING INTERESTS

The authors declare they have no competing interests.

## AUTHORS’ CONTRIBUTIONS

BG, VGV, BH, CP, TST, MB and GOM conceived and designed the ImPrEP CAB Brasil study. EC, CJ, NF and CGM conducted qualitative assessments. CP and CGM supervised qualitative data collection and performed data analysis. CP and TST drafted the manuscript. All authors critically reviewed and approved the final manuscript.

## FUNDING

TST was financed in part by Conselho Nacional de Desenvolvimento Científico e Tecnológico (CNPq; #311871/2021‐6) and Fundação Carlos Chagas Filho de Amparo à Pesquisa do Estado do Rio de Janeiro (FAPERJ #E‐26/201.270/2022). BG was financed in part by CNPq (#313265/2023‐2) and FAPERJ (#E.26/200.946/2022).

## Supporting information




**Supporting Information file 1**: SuppInfo1
Print of the initial screen of the mHealth videos with links

COREQ (COnsolidated criteria for REporting Qualitative research) Checklist

**Protocol Appendix E2** ‐ Participant Qualitative Interviews on PrEP Education, Choice and mHealth

## Data Availability

The data that support the findings of this study are available on request from the corresponding author. The data are not publicly available due to ethical restrictions.
