# Supplementary material for: Exploring perceptions and preferences for PrEP choice and of an mHealth intervention: insights from the ImPrEP CAB‐Brasil study
Source: J Int AIDS Soc. 2025 Jul 2;28(Suppl 2):e26493. doi: 10.1002/jia2.26493 (PMC12215825; doi:10.1002/jia2.26493)
Supplement: Supplementary file 1 — Supporting Information file 1: SuppInfo1 Print of the initial screen of the mHealth videos with links [file JIA2-28-e26493-s001.pdf]

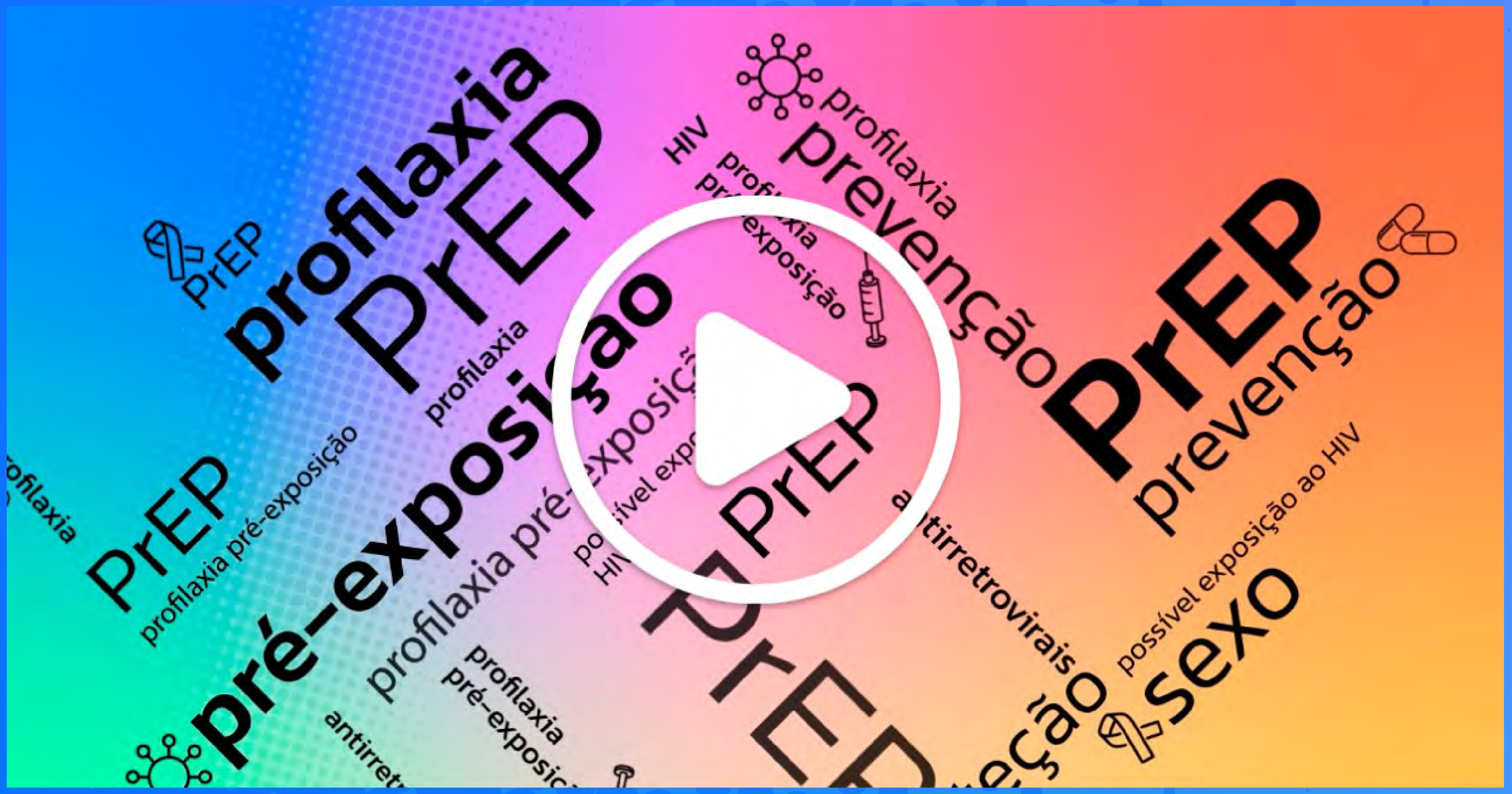

1 | Você conhece a PrEP oral?

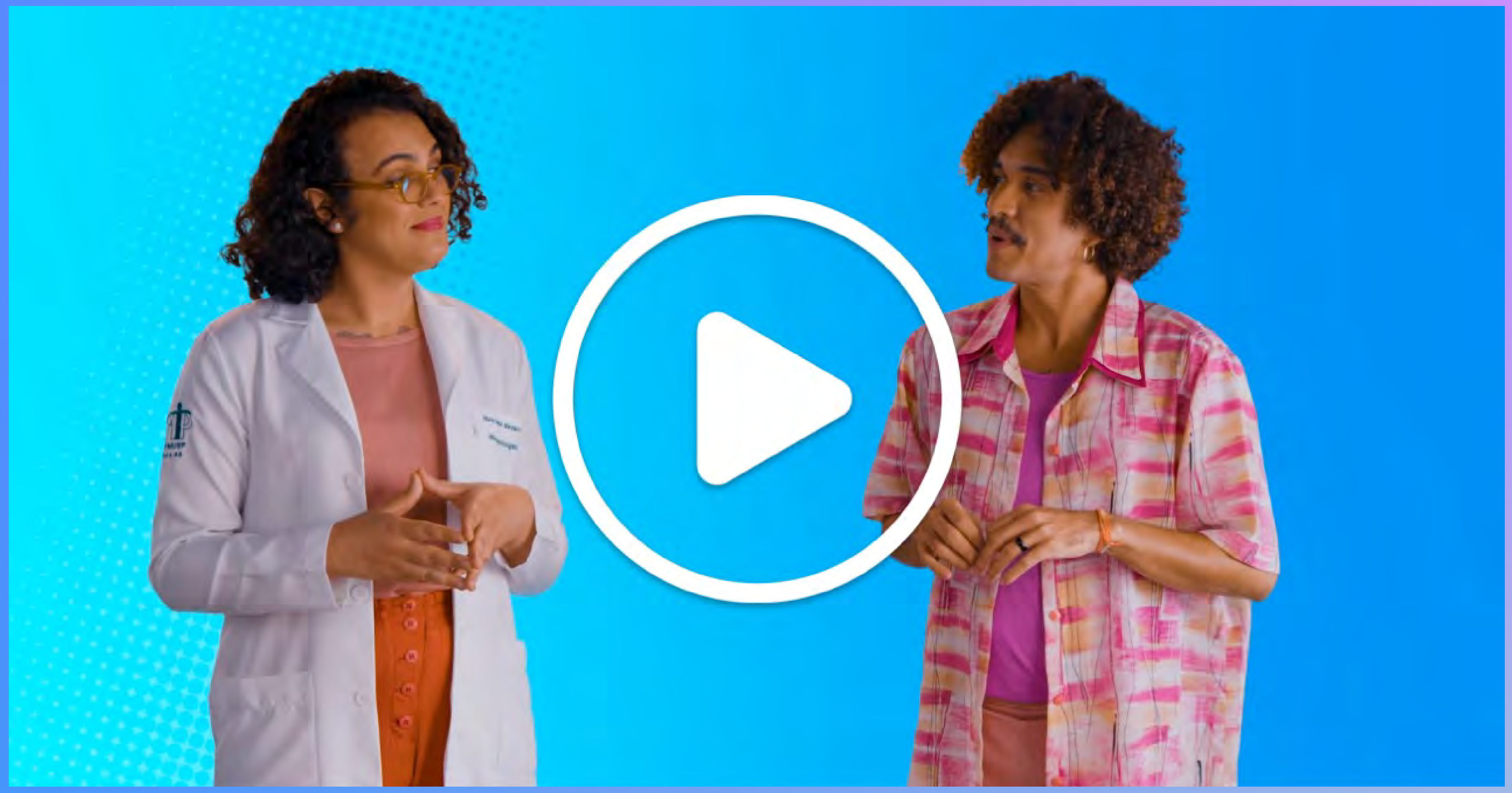

2 | Um nova forma de se proteger do HIV: PrEP injetável

QUAL FORMA DE PREVENÇÃO SE ADEQUA MELHOR À SUA ROTINA?

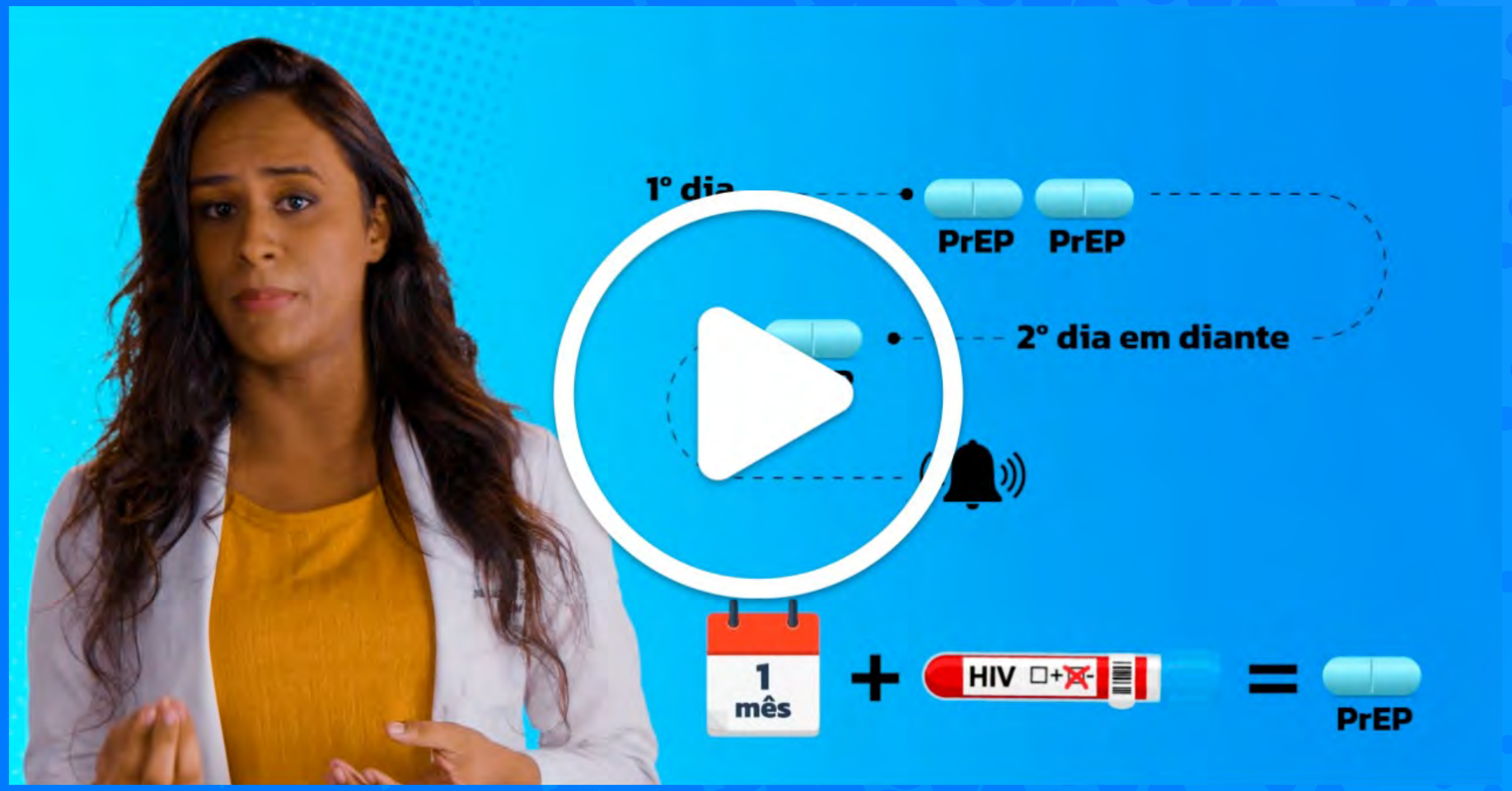

3 | Escolho PrEP oral

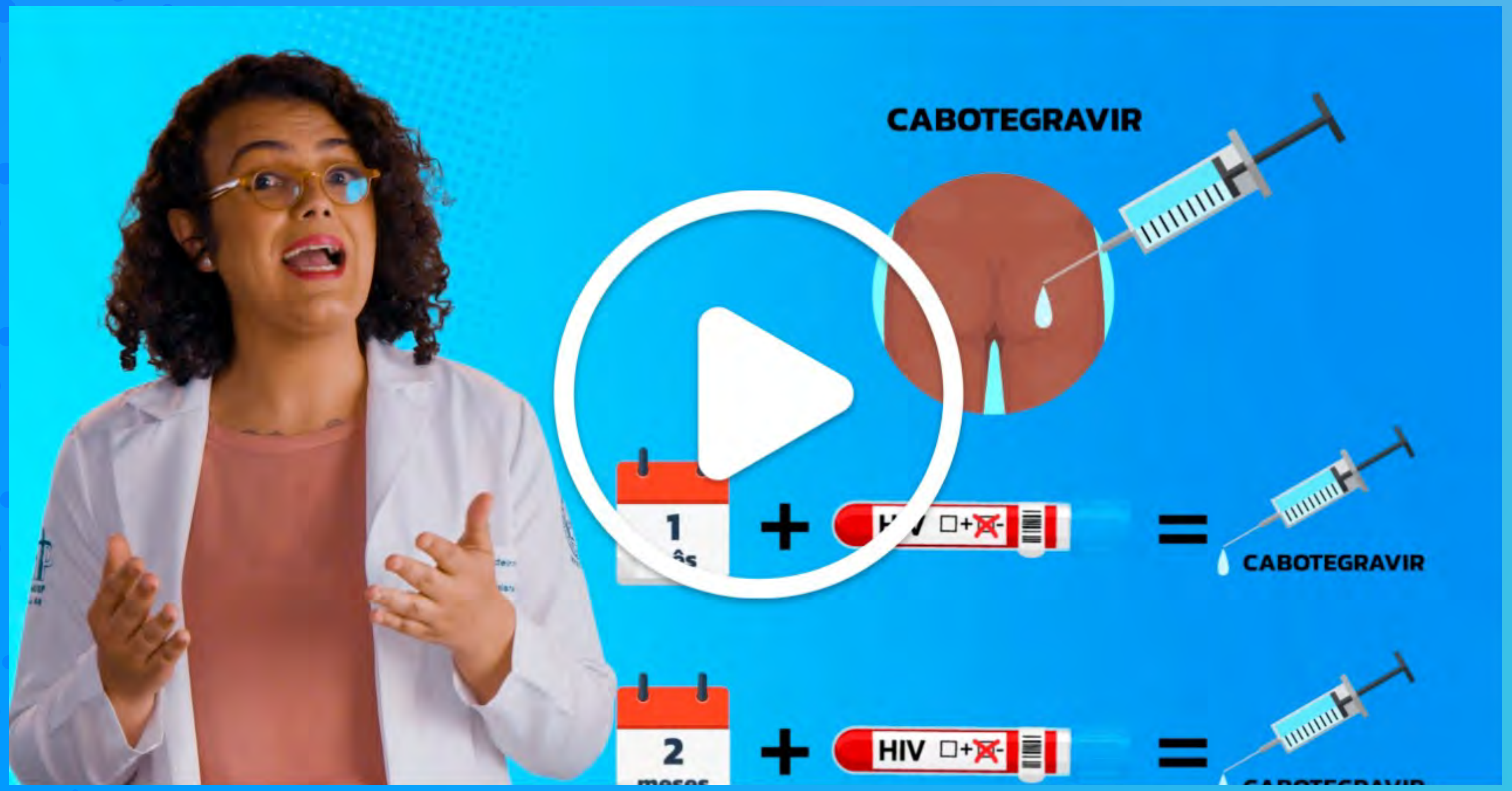

4 | Escolho PrEP injetável

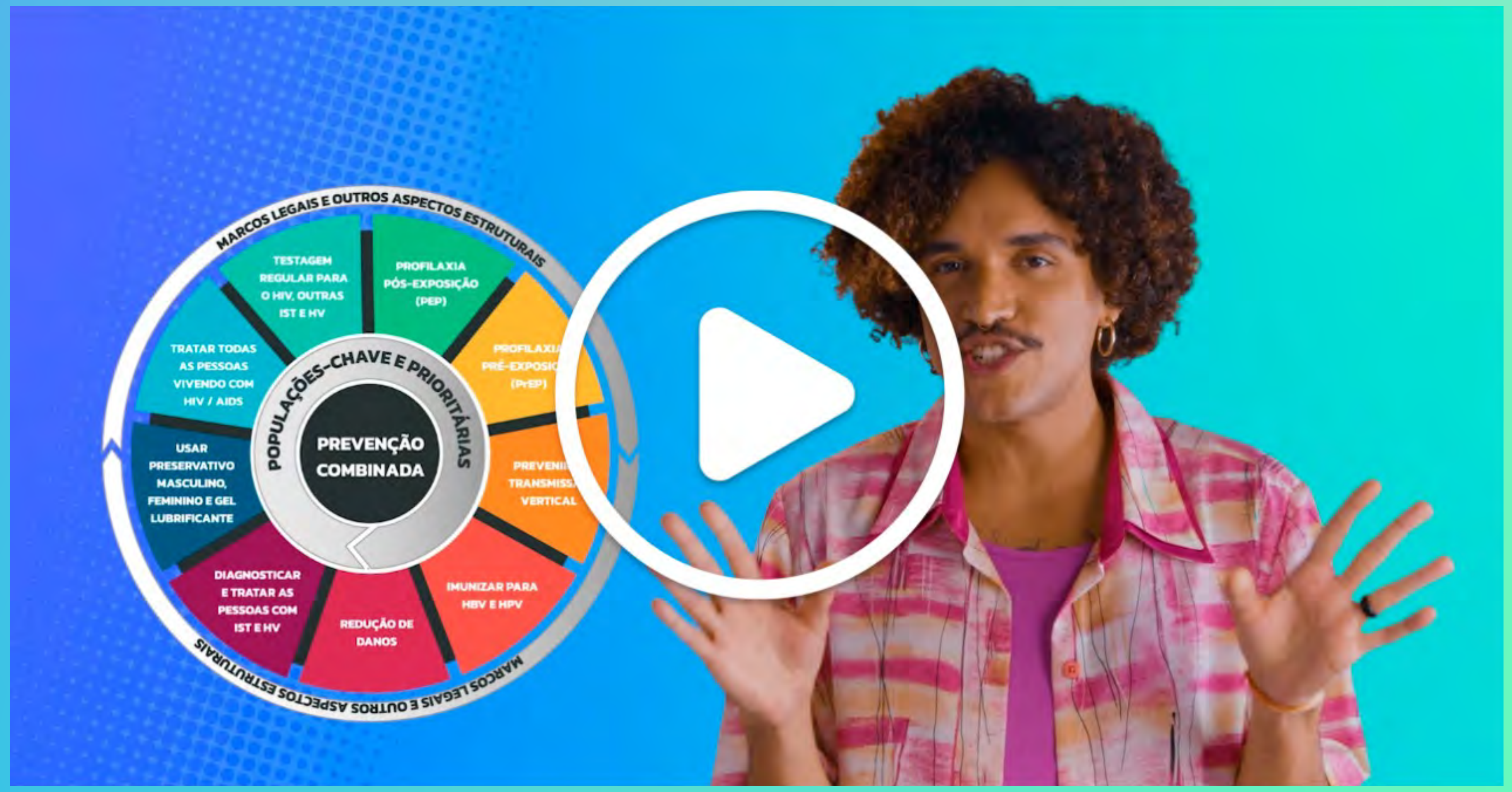

5 | Escolho outras formas de prevenção
