# Supplementary material for: Exploring perceptions and preferences for PrEP choice and of an mHealth intervention: insights from the ImPrEP CAB‐Brasil study
Source: J Int AIDS Soc. 2025 Jul 2;28(Suppl 2):e26493. doi: 10.1002/jia2.26493 (PMC12215825; doi:10.1002/jia2.26493)
Supplement: Supplementary file 3 — Protocol Appendix E2 ‐ Participant Qualitative Interviews on PrEP Education, Choice and mHealth [file JIA2-28-e26493-s003.pdf]

Protocol Appendix E2 - Participant Qualitative Interviews on PrEP Education, Choice and mHealth

Participant Qualitative Interviews on PrEP Education, Choice and mHealth
STEP 1

Interviewer instructions: The goal of these questions is to gather qualitative information about why participants choose either oral or CAB LA PrEP. Part A is to ask all participants who agree to be interviewed. Part B is only for participants who were exposed to the mHealth intervention.

PARTICIPANT NUMBER:
DATE (DD/MM/YY):
START TIME:
END TIME:
INTERVIEWER INITIALS:

Hello, good morning /or afternoon,
Thank you for agreeing to speak with me today. I work for the ImPrEP CAB LA Brazil implementation study.

As you know, we now have two types of PrEP available for HIV prevention.
We are interested in learning about why some people prefer PrEP injections and others prefer oral PrEP. We are also interested in hearing from you about how PrEP education and counselling practices could be improved. There are no right or wrong answer to any of these questions; we are really interested in hearing your perspectives. If you do not want to answer any of these questions, please let me know and we will go on to the next question. I think this interview will last about 20 minutes.

PART A: (1st visit - participants who received SOC)

1) Had you heard about oral PrEP and injectable PrEP before you came to the clinic today? If yes, where did you learn about these HIV prevention methods?

> Probe: if participant mention's internet, explore which online tools, e.g. social media (Facebook, Instagram, TikTok, Twitter, etc), dating apps (Grindr, Hornet, Scruff, Tinder, Happn, etc), YouTube; if participant mention influencers, try to get the names; if mention gay/LGBTQIAP+ venues/parties, explore type and name of these venues/parties.

2) When the health care provider talked with you today about your PrEP options, was there anything you did not understand, or that remained confusing? If so, what?

3) After you consulted with the health care provider about the HIV prevention options available to you, did you still have some questions? If so, what were they?

4) After talking with the health care provider, which HIV prevention method would you like to receive today? Why?

5) What do you think will be the most challenging aspect for you to continue using this method?

6) Do you have any concerns about using [whichever method participant chooses in Answer 4]? If yes, what are they?

PART B: (1st visit - ONLY for PARTICIPANTS who receive the mHEALTH INTERVENTION)

7) Did you appreciate/like learning about PrEP methods on the tablet before you went to talk to the health provider? Why or why not?

Probe: What did you like about the mHealth intervention? What did you not like about the mHealth intervention?

8) Do you think that all people coming for oral PrEP or for a PrEP injection should have the chance to learn about PrEP using the mHealth/tablet? Why or why not?

Probe: Which populations would benefit more? Which populations may have difficulties using the mHealth tool?

9) Did the mHealth intervention help you acquire information and decide whether to use oral PrEP or injectable PrEP? How?

10) What do you think should be done to make the mHealth intervention easier to use or more beneficial to helping people decide which HIV prevention method will be best for them?

11) Today, you have heard about CAB LA PrEP at the health clinic from the health provider. What suggestions do you have for how we should share/disseminate information about CAB LA PrEP to others in the community? (i.e. social media, apps, youtube, radio, billboards, etc).

12) What do you think is the most important thing for us to include in those informational/educational messages?

Do you have anything else you would like to say?
Thank you again for answering these questions
